# Prevalence and Characterization of PVL-Positive *Staphylococcus aureus* Isolated from Raw Cow’s Milk

**DOI:** 10.3390/toxins14020097

**Published:** 2022-01-25

**Authors:** Asmaa Sadat, Radwa Reda Shata, Alshimaa M. M. Farag, Hazem Ramadan, Adel Alkhedaide, Mohamed Mohamed Soliman, Mohamed Elbadawy, Amira Abugomaa, Amal Awad

**Affiliations:** 1 Department of Bacteriology, Mycology, and Immunology, Faculty of Veterinary Medicine, Mansoura University, Mansoura 35516, Egypt; asmaasadat@mans.edu.eg; 2 Department of Food Hygiene and Control, Faculty of Veterinary Medicine, Mansoura University, Mansoura 35516, Egypt; radwa.reda@yahoo.com; 3 Department of Internal Medicine and Infectious Diseases, Faculty of Veterinary Medicine, Mansoura University, Mansoura 35516, Egypt; dr_alshimaafarag@mans.edu.eg; 4 Hygiene and Zoonoses Department, Faculty of Veterinary Medicine, Mansoura University, Mansoura 35516, Egypt; hazem_hassan@mans.edu.eg; 5 Clinical Laboratory Sciences Department, Turabah University College, Taif University, Taif 21995, Saudi Arabia; a.khedaide@tu.edu.sa (A.A.); mmsoliman@tu.edu.sa (M.M.S.); 6 Department of Pharmacology, Faculty of Veterinary Medicine, Benha University, Moshtohor, Toukh 13736, Elqaliobiya, Egypt; Mohamed.elbadawy@fvtm.bu.edu.eg; 7 Faculty of Veterinary Medicine, Mansoura University, Mansoura 35516, Egypt

**Keywords:** *Staphylococcus aureus*, *pvl* gene, MRSA, antimicrobial resistance, enterotoxins

## Abstract

The present study aimed to investigate the prevalence, antibiotic susceptibility profiles, and some toxin genes of Panton-Valentine leukocidin (PVL)-positive *Staphylococcus aureus* (*S. aureus*) in unpasteurized raw cow’s milk collected from retail outlets located at Mansoura, Dakahliya governorate, Egypt. In that context, a total of 700 raw cow’s milk samples were investigated for the presence of *S. aureus*, which was identified in 41.1% (288/700) of the samples. Among the *S. aureus* isolates, 113 PVL-positive *S. aureus* were identified and subjected for further analysis. The PVL-positive *S. aureus* were investigated for the existence of toxin-related genes, including hemolysin (*hla*), toxic shock syndrome toxin-1 (*tst*), and enterotoxins (*sea*, *seb*, *sec*, *see*, *seg*, *sei*, and *selj*). Genotypic resistance of PVL-positive strains was performed for the detection of *blaZ* and *mecA* genes. Among the PVL-positive *S. aureus*, *sea*, *seb*, and *sec* were detected in 44.2, 6.2%, and 0.9%, respectively, while the *hla* and *tst* genes were identified in 54.9% and 0.9%, respectively. The *blaZ* and *mecA* genes were successfully identified in 84.9 (96/113) and 32.7% (37/113) of the total evaluated *S. aureus* isolates, respectively. PVL-positive *S. aureus* displayed a high level of resistance to penicillin, ampicillin, and trimethoprim-sulfamethoxazole. Multidrug resistance (resistant to ≥3 antimicrobial classes) was displayed by all methicillin-resistant *S. aureus* (MRSA) and 38.2% of methicillin-sensitive *S. aureus* (MSSA) isolates. The obtained findings are raising the alarm of virulent PVL-positive MRSA clones in retail milk in Egypt, suggesting the requirement for limiting the use of β-lactam drugs in food-producing animals and the importance of implementing strong hygiene procedures in dairy farms and processing plants.

## 1. Introduction

Milk and dairy products are significant sources of macro-and micro-nutrients required by humans, making them vulnerable to microbial pathogen contamination. The ingestion of contaminated raw milk is the major cause of serious food-poisoning outbreaks, potentially resulting from microbial toxin production [1]. Contaminated raw milk may contain hazardous microorganisms that cause milk to spoil or the onset of public health hazards [2]. *Staphylococcus aureus* (*S. aureus*) is one of the most important opportunistic pathogens of raw milk that can cause serious infection in humans [3].

*Staphylococcus aureus* has high pathogenicity due to its widespread distribution, high contamination rate, and rapid transmission. It can cause a wide range of clinical symptoms, from minor superficial skin lesions to significant invasive infections, and it can even be life-threatening [4]. However, *S. aureus* can enter milk through direct excretion from the udder of a cow with clinical or subclinical staphylococcal mastitis, as well as through contamination from the environment during raw milk handling and processing, posing a risk to consumers [5]. Therefore, it is critical to monitor virulent strains of *S. aureus* regularly to detect the potential risk this bacterium poses to public health [6].

The severity of the clinical signs and infections caused by *S. aureus* are linked to the presence of virulence factors. Among them, extracellular toxins, including staphylococcal enterotoxins (SEs), toxic-shock syndrome toxin-1 (TSST-1), staphylococcal hemolysins, and Panton-Valentine leukocidin (PVL) [7,8,9]. These toxins are the cause of food poisoning and many other clinical manifestations affecting humans and animals. Staphylococcal enterotoxins (SEs) are thermostable toxins that cause enterotoxaemia due to the consumption of food contaminated with enterotoxigenic strains [10]. Staphylococcal enterotoxins (SEs) act as superantigens that trigger the expression of the *IL-4* and *IL-10* genes, followed by the activation of TH2 cells. As a result, the clearance of invading infections is suppressed [11]. Many SEs have been discovered, and classical enterotoxins are divided into five serological types: SEA, SEB, SEC, SED, and SEE [12]. The traditional enterotoxin genes have been responsible for 90% of food poisoning outbreaks [13]. Among the Staphylococcus virulence factors, toxic shock syndrome toxin TSST-1 is a superantigenic and powerful toxin that causes toxic shock syndrome, which is characterized by many clinical signs, multi-organ dysfunction, and, finally, death. TSST-1 activates T cells in a nonspecific manner, resulting in an excessive immune response and excessive cytokine production [14]. Additionally, *S. aureus* produces alpha, beta, gamma, and delta hemolysin. Among them, alpha-toxin (Hla) is produced by most pathogenic strains and is considered a major virulence factor [15]. Several studies have reported the roles of α-hemolysin in *S. aureus* pathogenesis, including cytokine release, cell signaling pathways that control cell proliferation, inflammatory responses, and cell–cell interactions, which result in mammary gland necrosis and higher mortality rates among the infected animals [16,17,18,19].

Panton-Valentine leukocidin toxin is a potent staphylococcal exotoxin that is activated by two secretory proteins of the F and S kinds [20]. It acts strongly on human polymorphonuclear cells and causes severe clinical manifestations, particularly with methicillin-resistant *S. aureus* (MRSA), such as furunculosis, severe necrotizing pneumonia, and skin and tissue necrosis [21]. In patients with necrotizing pneumonia, it has been observed that the risk of death linked with *S. aureus* strains expressing the *pvl* gene is higher than that associated with PVL-negative bacteria [22].

An important public health issue concerning staphylococcal infections is the development of antibiotic resistance. Antibiotic resistance has emerged as a result of the widespread use of antimicrobials in food animal production, reducing the efficacy of various antibiotic classes for the treatment of infections in both humans and animals, particularly β-lactam antimicrobials, which are the most commonly used antibiotics in the treatment of animal diseases [23]. The use of these antibiotics in subtherapeutic doses in developing countries, including Egypt, for growth promotion and disease prevention raises the danger of new and more resistant bacteria emerging [24]. *S. aureus* resistance to β-lactam antibiotics is caused by two mechanisms: the production of penicillinase encoded by the *blaZ* gene [25], which is involved in the hydrolysis of β-lactam and inactivation of antibiotic, and an altered low-affinity penicillin-binding protein (PBP2-a) encoded by *mecA,* which accounts for methicillin-resistance [26,27,28].

Methicillin-resistant *S. aureus* (MRSA) with the *mecA* gene is resistant to a variety of antimicrobials [29]. Contamination of food by multidrug-resistance MRSA strains with a wide range of exotoxins, including enterotoxins and *pvl* genes, confers life-threatening traits on MRSA, thereby making treatment complicated.

In Egypt, loose liquid milk sold at retail stores and by street vendors is preferred by consumers over bottled milk, which is thought to be made with powder milk, making it less appealing. There are several Egyptian reports regarding the prevalence and characterization of *S. aureus* possessing the *pvl* gene in clinical isolates [30,31,32,33]. Despite its role in various severe clinical manifestations, genotypic and phenotypic analyses of PVL-positive *S. aureus* in retail food are still poorly performed in Egypt. Considering that raw milk is a vehicle for the transmission of numerous bacteria, including toxigenic and multidrug-resistant strains of human and animal origin, and represents a great risk for both animal and public health, this investigation aimed to find out the prevalence of PVL-positive *S. aureus* in retail milk and to characterize the obtained strains for their phenotypic and genotypic antimicrobial susceptibility and virulence traits to recognize the genetic background of food-related PVL-positive *S. aureus* in Egypt.

## 2. Results

### 2.1. Prevalence and Molecular Characterization of PVL-Positive S. aureus

**Figure 1:**
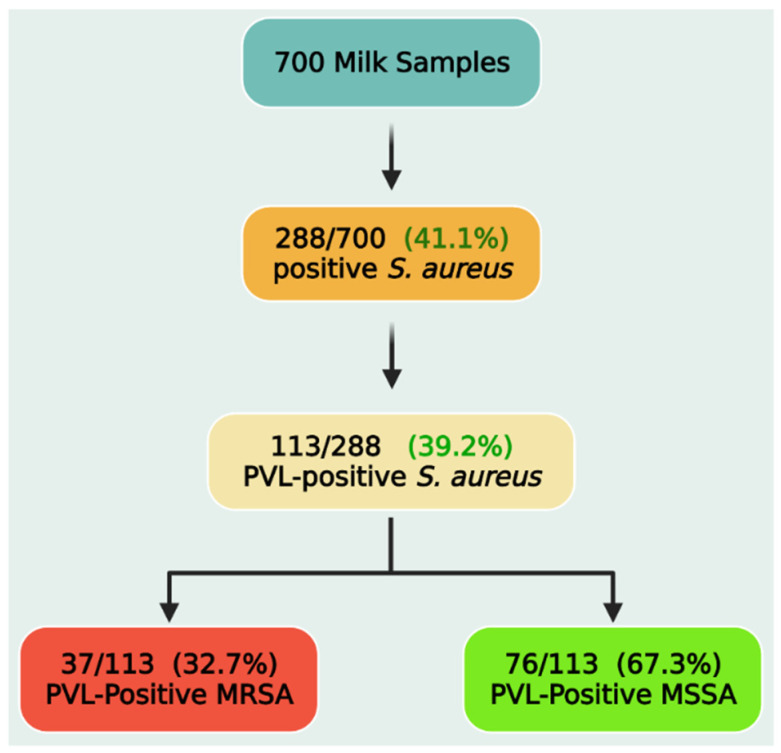
Prevalence of PVL-positive *Staphylococcus aureus*, methicillin-resistant (MRSA), and methicillin-sensitive *Staphylococcus aureus* (MSSA) strains.

Out of the investigated retail milk samples (*n* = 700), 288 isolates were biochemically identified as *S. aureus*. The primer set used in this study for detection of the *nuc* gene (encodes a thermonuclease) yielded the expected 660-bp product for that gene and succeeded to amplify this product for all the tested isolates with an overall prevalence of 41.1% (288/700). Subsequently, *S. aureus* isolates were tested for the presence of the *pvl* gene. Overall, of the 288 *S. aureus* isolates, 113 (39.2%, 113/288) tested positive for the *pvl* gene (Figure 1). As a result, 113 bacterial isolates were subjected to genotypic and phenotypic analyses. PVL-positive *S. aureus* were then screened for the presence of the *mecA* gene, which was detected in 37 isolates (32.7%), and they are classified as PVL-positive methicillin-resistant *S. aureus* (MRSA), while *mecA*-negative isolates were classified as PVL-positive methicillin-sensitive *S. aureus* (MSSA).

### 2.2. Virulence Gene Profiles and Genotypic Profiles of β-Lactam Resistance

PVL-positive *S. aureus* isolates were screened for the presence of toxin gene markers. In total, 50.4% (57/113) of PVL-positive *S. aureus* were found to harbor one or more SEs gene. In detail, the *sea* gene was the most common enterotoxin gene found among the tested isolates (50/113; 44.2%), followed by the *seb* gene (7/113; 6.2%) and the *sec* gene (1/113; 0.9%), while *see*, *seg*, *sei*, and *selj* were not detected in this study. In addition, the *hla* and *tst* genes were found in 54.9% (62/113) and 0.9% (1/113), respectively (Table 1 and Table S1). PCR targeting the *blaZ* and *mecA* genes was used to test *S. aureus* strains for their genotypic resistance. Both the *blaZ* and *mecA* genes were recognized in 84.9% (96/113) and 32.7% (37/113) of the PVL-positive *S. aureus* isolates, respectively (Table 1 and Table S1).

### 2.3. Antimicrobial Susceptibility Testing and Resistance Patterns of the PVL-Positive MRSA and MSSA

The results of antimicrobial susceptibility testing of PVL-positive *S. aureus* isolates (*n* = 113) are listed in Table 2. The PVL-positive *S. aureus* isolates were highly resistant to penicillin, ampicillin, and trimethoprim-sulfamethoxazole (90.3%, 70.8%, and 54.9%, respectively), and moderately resistant to ciprofloxacin (41.6%), clindamycin (37.2%), and erythromycin (34.5%). However, they were more sensitive to gentamicin (79.6%) and chloramphenicol (85.8%).

A significantly higher resistance rate was displayed by the MRSA isolates (*mecA*-positive) to all antimicrobials than the MSSA isolates (*mecA*-negative). All MRSA isolates (100%) were resistant to penicillin and ampicillin, while 94.6% (35/37) were resistant to trimethoprim-sulfamethoxazole, 64.9% (24/37) to ciprofloxacin, 59.5% (22/37) to erythromycin, 54.1% (20/37) to clindamycin, 86.5% (32/37) to tetracycline, 32.4% (12/37) to chloramphenicol, and 29.7% (11/37) to gentamycin. Interestingly, all MRSA isolates displayed MAR (resistance to three or more antimicrobial classes). Notably, 6.58% (5/76) of methicillin-sensitive *S. aureus* (MSSA) isolates were susceptible to all tested antimicrobials, 55.3% (42/76) of the 76 isolates were resistant to at least 1 antibiotic, and 38.2% (29/76) were resistant to 3 or more antimicrobial classes. The most prevalent antimicrobial-resistant profile of the MRSA isolates was P, AMP, CIP, DA, E, SXT, and TE. A higher MAR index was also noticed among MRSA isolates, which ranged between 0.44 and 1.0, while in MSSA it ranged between 0.00 and 1 (Table 3 and Table 4, respectively).

### 2.4. Association of Resistance Phenotypes, Resistant Genes, and Virulence-Associated Genes in PVL-Positive S. aureus

**Figure 2:**
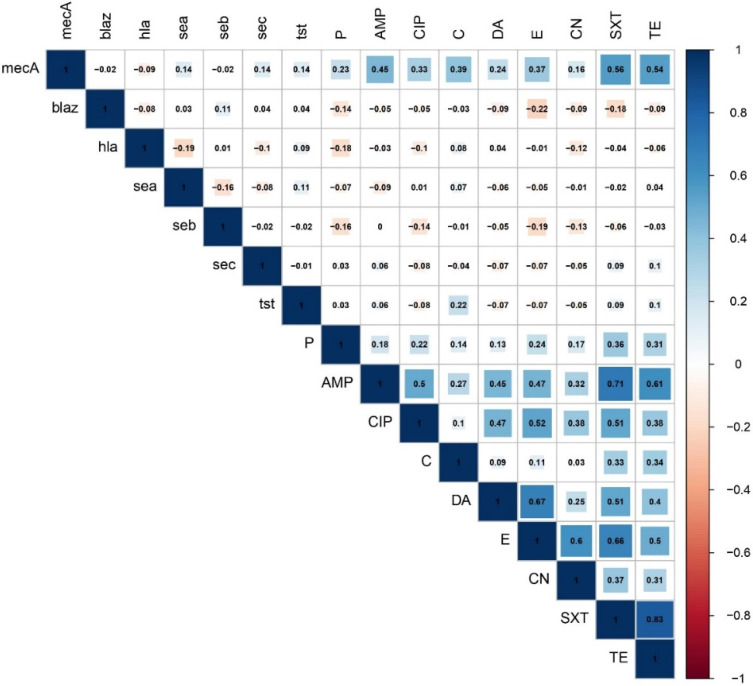
Association of resistance phenotypes, resistance genes, and virulence-associated genes in PVL-positive *Staphylococcus aureus*. The blue and orange colors of the boxes indicate positive and negative correlations, respectively. The strength of the colors corresponds to the numerical value of the correlation coefficient (r).

The findings of correlation analysis revealed the existence of significant (*p* < 0.05) positive associations between pairs of antimicrobials belonging to different antimicrobial classes (Figure 2 and Figure S1). Strong positive correlations (r > 0.6) were observed between the following antimicrobial pairs: TE and SXT (*r* = 0.83), SXT and AMP (*r* = 0.71), DA and E (*r* = 0.67), SXT and E (*r* = 0.66), and TE and AMP (*r* = 0.61). Moderate (*r* = 0.4–0.6) and weak positive (r < 0.4) correlations were also determined between pairs of the tested antimicrobials (Figure 2). Of the two resistance genes examined, the *mecA* gene showed significant moderate correlations with SXT (*r* = 0.56), TE (*r* = 0.54), and AMP (*r* = 0.45) and weak positive correlations with C (*r* = 0.39), E (*r* = 0.37), CIP (*r* = 0.33), and DA (*r* = 0.24). Concerning the virulence–resistance relationship, no significant correlations were found between the examined resistance and virulence genes.

## 3. Discussion

Raw milk may pose a consumer risk, due to the possible presence of human pathogenic bacteria, such as *S. aureus*. *S. aureus* has been identified as a major cause of zoonotic disease, with the potential for MRSA transmission between animals and humans by direct contact, handling, and/or eating of *S. aureus*-infected animal products [34]. This investigation was conducted on a total of 700 raw milk samples, and a high prevalence (41.1%) of *S. aureus*-contaminated raw cow’s milk was found among the total examined samples. This finding is in agreement with those of other investigations performed in Egypt, which were ranged from 35.9 to 75% [11,35,36,37,38,39], in Turkey, where a 56% prevalence rate of *S. aureus* was recovered out of 150 raw milk samples [40], and in China, where, out of 195 samples, 54 (27.7%) were positive [41] and out of 144 samples, 62 (43.1%) were positive for *S. aureus* [42].

In Egypt, the production and consumption of milk and dairy products are regulated by the law 132/1950, which consists of 14 articles. Moreover, the Egyptian general organization for standards and quality (EOS) issued a set of standards (154-1/2005) for milk and milk products (part1: raw milk) [43], available at: https://www.eos.org.eg/en/standard/2484, accessed on 20 December 2021. Generally, all dairy products (yogurt, cream, and butter) must be prepared from pasteurized milk, and all ingredients used in their manufacture must conform to the standard specifications of each of them. The Egyptian government allows the sale of raw (unpasteurized) milk in small shops and supermarkets. However, it must comply with the rules of the law and standards which set several standards regarding the dairy animals, milk and milk products, milk utensils, and milk handlers. Accordingly, the sale of milk is prohibited unless it is from healthy animals, clean and fresh, retains all its natural properties, free from impurities, dirt, and colored materials (Article 2). Further, milk must be free from several pathogens (specified in article 3) such as *Brucella*, *Listeria monocytogenes*, *Mycobacterium tuberculosis*, *Bacillus anthracis*, and *Salmonella*, while total *Clostridium perfringens* and *Bacillus cereus* must be <1 colony-forming unit (cfu)/mL), and total *S. aureus* count must be <100 cfu/mL (ES, 2005) [43]. The law also prohibits the sale or use of milk from some diseased animals (article 3), sets requirements for transportation means (article 6), milk containers (article 7), milk handlers, and street vendors allowed to sell milk (article 9).

Contamination of raw milk with *S. aureus* is usually related to mastitis or human carriers. The main sources of raw milk contamination include the milking process, environmental contamination with diseased animal manure, and inadequate handling during transportation to outlets and at milk collection facilities [44]. Therefore, raw milk collection, production, transportation, and sale should all be standardized. Simultaneously, appropriate professional training for workers is essential to reduce raw milk pollution caused by poor hygiene. PVL-positive *S. aureus* is a highly pathogenic *S. aureus* strain carrying PVL that affects humans and causes recurrent and potentially serious infections of the skin, ranging from isolated recurrent abscesses to extensive furunculosis and necrotizing pneumonia [45]. Delayed identification of PVL-positive *S. aureus* can lead to serious morbidity and mortality ranges from 40 to 60% [46]. In this study, PVL-positive *S. aureus* strains were found in 39.2% of all *S. aureus* isolates tested. The detection of PVL-positive *S. aureus* from bovine milk with varying percentages has been reported in Korea [47], Minnesota (USA) [48], and Jabalpur (India) [49]. The frequency of PVL-positive *S. aureus* varies, depending on the sample type, geographic area [6], disparities in livestock breeding systems, animal species, milking methods, and hygienic conditions [50]. As a result, raw milk collection, production, transportation, and sale should all be standardized.

Panton-Valentine leukocidin is a human-associated leukocidin found mostly in *S. aureus* strains from humans [51,52]. The isolation of this gene from milk samples suggests that *S. aureus* is transmitted from human to cow [14,53,54]. The introduction of this bacterium from milkers’ hands or teat cup liners to the udder may have contributed to poor hygiene during the milking process. As a result, because it is a food-related pathogen, it must be regarded a possible public health danger.

*Staphylococcus aureus* produces various virulence factors in addition to PVL. These factors are important for evading host defenses and causing microbial colonization of the animals’ udders. Exotoxins produced by *S. aureus* can cause epidemics of staphylococcal food poisoning (SFP) from milk and dairy products intended for human consumption [55,56]. The pore-forming cytotoxin hemolysin, which is one of the key virulence factors of *S. aureus* [57], was found in 54.9% of the PVL-positive *S. aureus* in this study. Similarly, Al-ashmawy et al. [36] found that the α-hemolysin encoding gene (*hla*) was the most often discovered virulence gene from raw milk in Egypt (100%) and in China (Ren et al. [58]; 96.9%). It has been reported that staphylococcal enterotoxins (SEs) can retain their biological and immunological effects, even after pasteurization. The potential hazard posed by these strains was highlighted in several studies [51,59]. The classical enterotoxin genes were responsible for 90% of food poisoning occurrences [13]. In this investigation, at least one kind of enterotoxin gene was found in 57 (50.44%) of PVL-positive *S. aureus* isolates. The *sea* gene was the most often encountered gene in this study, followed by the *seb* and *sec* genes. These findings are consistent with those of Chao et al. [60] and Peles et al. [61], who found a similar distribution of enterotoxin genes in *S. aureus* isolates in China and Hungary, respectively, while the *sea*, *seb*, and *see* genes were not identified. In addition, only 5 isolates exhibited the *sec* gene with a frequency of 6% in milk obtained from cows with subclinical mastitis in Wisconsin [62]. The *sea* gene is very resistant to pasteurization heat and maintains some biological activity at high temperatures [63]. It was reported as the most frequently identified gene in US food poisoning outbreaks [64].

Toxic shock syndrome toxin-1 is one of the most important virulence factors of *S. aureus*, causing multi-organ malfunction and acting as a superantigen, contributing to the pathogenic processes of bovine mastitis [65]. In our investigation, the *tst* gene, which is responsible for the production of TSST-1, was found infrequently (0.9%). In much prior research, *tst* was also found in a small percentage of *S. aureus* isolates from bovine milk [37,49,66,67], while Wang et al. [15] and Ren et al. [58] found a greater frequency of *tst* (94% and 26.2%, respectively).

Out of 113 PVL-positive *S. aureus* isolates, 32.7% were confirmed as MRSA by the detection of *mecA*. The presence of MRSA in the examined PVL-positive isolates is a major public health concern due to the possibility of the transmission of multidrug-resistant MRSA strains through the food chain. A foodborne outbreak caused by MRSA has been reported previously [68]. PVL-positive MRSA was also detected in raw milk in Egypt in several previous studies [36,37,69,70,71], while, in other studies, none of the MRSA isolated from food harbored the *pvl* gene [66,71,72]. Acquisition of the *pvl* genes by MRSA is considered a problem for controlling infection by this clone. On the other hand, in this investigation, it was discovered that the presence of the *mecA* gene was not linked to the existence of enterotoxins in the tested isolates.

Antimicrobial agents, particularly β-lactams, are used worldwide to control *S. aureus* infection [73]. In rural areas, after injection of antibiotics to dairy animals, some farmers do not follow the guidelines regarding drug residues and milk their cows and sell the milk to the retail markets [74,75,76,77]. Unfortunately, no legislation in Egypt controls the use of antibiotics in dairy animals, according to WHO reports in 2013 [78] and 2017 [79] and Tartor et al. [80]. However, several organizations in Egypt make periodical inspection visits to dairy farms and plants to collect samples of the dairy products for analysis to assure that they conform to standards. However, this is mostly for large companies, and the control is weak over small unregistered and unlicensed plants. In the present study, penicillin had a resistance rate of 90.3% (102/113), which was the highest rate among the PVL-positive *S. aureus* isolates. These results agreed with many previous studies [49,81]. The *blaZ* gene, which is a common β-lactam resistant mechanism for *S. aureus*, was detected in 84.9% of the total examined strains. The high resistance against penicillin may be related to the frequent use of penicillin in the treatment of mastitis and the drying-off period. The phenotypic and genotypic differences in the results to penicillin agreed with Yang et al. [82] and Silva et al. [83], which may have contributed to the other resistance mechanisms or the expression of other genes. Additionally, some *blaZ* gene detection did not have a detectable phenotype, which may be related to the mechanisms of gene inactivation [84].

The high prevalence of MAR *S. aureus* and the presence of methicillin resistance among *S. aureus* are of both clinical and public health concerns. Interestingly, all MRSA isolates displayed MAR (resistance to three or more antimicrobials). While 6.58% of MSSA isolates were susceptible to all tested antimicrobials, 55.3% were resistant to at least 1 antibiotic and 38.2% were multidrug-resistant to 3 or more antimicrobial classes. The presence of a high percentage of MAR *S. aureus* isolated from dairy cattle has been reported in Ethiopia (98.3%), Italy (50%), and South Africa (62%) [85,86,87]. In the present study, penicillin resistance was common among MAR *S. aureus* isolates, which was also reported in several studies [88,89]. Interestingly, the values of the MAR index for all MRSA and most MSSA strains were higher than 0.2, suggesting that the origin of these strains is from a high-risk source of contamination where antibiotics are frequently used. These findings underline the importance of adopting control measures against *S. aureus* in dairy farms to minimize the risks for both animal and public health.

## 4. Conclusions

The obtained results provide information regarding the prevalence of *S. aureus* from retail milk in Egypt. The isolated strains carried toxin genes that had a virulence potential and potential health risks which necessitate proper hygiene practices to prevent the spread of this clone through the food chain. In addition, it is necessary to raise awareness of smallholders of the necessity to keep milk refrigerated after milking and during transportation to the retail outlets to prevent food from being held at an unsafe temperature and avoid food poisoning by *Staphylococcus*. Our findings also revealed the existence of an alarming level of MRSA strains, and the development of multidrug resistance indicates an alarming situation; therefore, it is necessary to continuously monitor the use of antibiotics in dairy herds to minimize the risks for both animal and public health. Future studies should be conducted to investigate the risk involved in dairy cow colonization/infection.

## 5. Materials and Methods

### 5.1. Sampling

Unpasteurized raw cow’s milk samples (*n* = 700) were collected from retail outlets located in Mansoura, Dakahliya, Egypt. Raw milk was obtained from retail markets which receive raw milk daily from smallholders who depend totally on hand milking. Fresh milk is transported to retail shops without refrigeration during transportation in small tanks. In retail shops, milk is dispensed in little plastic bags that are kept refrigerated until sold to the consumers. Samples were collected weekly from 20 retail markets from February 2019 to March 2020. The samples were stored in an insulated box containing ice packs (precooled to −20 °C for 24 h) and transported immediately to the laboratory of the Bacteriology, Mycology and Immunology department, Faculty of Veterinary Medicine, Mansoura University for examination within 3 h.

### 5.2. Bacterial Isolation

Milk samples were incubated at 37 °C for 24 h and then centrifuged at 3000 RPM for 5 min. The cream layer was discarded and the sediments were streaked on a mannitol salt agar (MSA) medium (Oxoid Ltd., Hampshire, England) and incubated for 24 h at 37 °C to isolate *S. aureus*. The *S. aureus* were initially identified based on colony features (yellow colonies on MSA). One suspicious colony was picked up from MSA to be purified on trypticase soya agar (TSA, Oxoid Ltd., Hampshire, England), and kept at 4 °C for gram staining and biochemical assays (catalase, oxidase, and the tube coagulase test) [56]. The purified *S. aureus* colonies were preserved at −80 °C in 30% glycerol for further molecular characterization.

### 5.3. Genomic DNA Extraction and Molecular Characterization of PVL-Positive S. aureus

Single colonies from an overnight culture were suspended in 1 mL of distilled water, homogenized by vortexing, and centrifuged for 1 min at 13,000 RPM. The bacteria were then resuspended in 200 μL of distilled water, heated for 10 min at 100 °C, and centrifuged again for 1 min at 13,000 RPM [14]. Thereafter, the obtained supernatant was stored at −20 °C for further molecular characterization. The species-specific *nuc* gene was used to confirm *S. aureus* by PCR (encoding for the *S. aureus*-specific thermonuclease) using the following primer sets: *nuc*-F: (GCGATTGATGGTGATACGGTT) and *nuc*-R: (AGCCAAGCCTTGACGAACTAA AGC) [90]. An initial denaturation at 94 °C for two min was applied, followed by 35 cycles of denaturation at 98 °C for 10 s, annealing at 58 °C for 30 s, and extension at 68 °C for 1 min, followed by a final extension at 68 °C for 7 min. Confirmed *S. aureus* (*nuc*-positive) isolates were subjected to PCR for amplification of the *pvl* gene, which was performed according to Lina et al. [46] using the primer sequences tabulated in Table 5 with the following cycling condition: 30 s of denaturation at 94 °C, 30 s of annealing at 55 °C, and 1 min of extension at 72 °C for 30 cycles. The PCR products were visualized by electrophoresis through 1% agarose stained by ethidium bromide. 

### 5.4. Antimicrobial Susceptibility Test

According to the Clinical and Laboratory Standards Institute (CLSI), PVL-positive *S. aureus* isolates were tested for their antimicrobial susceptibility using the Kirby Bauer disk diffusion method on Muller Hinton agar plates (MH, Oxoid). The antimicrobials chosen are representative of those used in animal medicine. The following antibiotic disks were selected: ciprofloxacin (CIP; 5 μg), erythromycin (E; 15 μg), gentamicin (CN; 10 μg), penicillin (P; 10 IU), clindamycin (CL; 2 μg), ampicillin (AMP; 10 μg), chloramphenicol (C; 30 μg), trimethoprim-sulfamethoxazole (SXT; 25 μg), and tetracycline (TE; 30 μg). The results were recorded and interpreted after 24 h of incubation at 37 °C following CLSI guidelines [91,92]. Multidrug resistance was mentioned as a single strain resistant to three or more antimicrobial classes [93]. A multiple antibiotic resistance (MAR) index was calculated by dividing the total number of antimicrobial resistances for each isolate by the total number of antimicrobials tested, according to Krumperman [94].

### 5.5. Detection of Virulence-Associated Genes

The PVL-positive *S. aureus* isolates were screened for the presence of several staphylococcal enterotoxin genes (SEs) by multiplex PCR. The detection of seven genes encoding enterotoxigenicity in PVL-positive *S. aureus* (*sea*, *seb*, *sec*, *see*, *seg*, *sei*, and *selj*) was performed using seven specific primer sets, according to Monday and Bohach [95] and Johnson et al. [96], while toxic shock syndrome toxin-1 (*tst*) and the hemolysin gene (*hla*) were investigated as previously described by Sallam et al. [90].

### 5.6. Genotypic Profile of β-Lactam Resistance

The PVL-positive *S. aureus* isolates were subjected to PCR for the detection of β-lactam resistance genotypes by amplification of *mecA,* which encodes the protein PBP2A, and *blaZ*, which encodes β-lactamases. For detection of both the *blaZ* and *mecA* genes, PCRs were performed according to Oliveira et al. [97]. In brief, PCRs were performed in a 96-well 2720 thermal cycler (Applied Biosystems, Norwalk, CT) with a total volume of 25 μL of reaction mix using the following cyclic conditions: initial denaturation at 95 °C for 5 min, followed by 30 cycles of 95 °C for 1 min, 54 °C for 1 min, 72 °C for 1 min, and a final extension at 72 °C for 7 min. An aliquot of about 5 μL of PCR product of each reaction was visualized by electrophoresis in 1% agarose stained by ethidium bromide.

**Table 5:** Sequences of primers and PCR conditions for the targeted genes examined in the present study.

Target Gene	Primer Direction and Sequence	Amplicon Size (bp)	Reference
*nuc*	F: GTGCTGGCATATGTATGGCAATTG	660	[90]
	R: CTGAATCAGCGTTGTCTTCGCTCCAA		
*Pvl*	F: ATCATTAGGTAAAATGTCTGGACA TGATCCA	433	[46]
	R: GCATCAACTGTATTGGATAGCAAAAGC		
*mecA*	F: TCCAGATTACAACTTCACCAGG	162	[97]
	R: CCACTTCATATCTTGTAACG		
*blaZ*	F: TACAACTGTAATATCGGAGGG	861	[97]
	R: CATTACACTCTTGGCGGTTTC		
*tst*	F: CGTAAGCCCTTTGTTGCTTG	543	[90]
	R: CCACCCGTTTTATCGCTTGAAC		
*hla*	F: CCGGTACTACAGATATTGGAAGC	744	[90]
	R: GGTAATCATCACGAACTCGTTCG		
*seb*	F: TCG CAT CAA ACT GAC AAA CG	478	[96]
	R: GCA GGT ACT CTA TAA GTG CC		
*sea*	F: GCA GGG AAC AGC TTT AGG C	520	[95]
	R: GTT CTG TAG AAG TAT GAA ACA CG		
*sec*	F: CTT GTA TGT ATG GAG GAA TAA CAA	283	[95]
	R: TGC AGG CAT CAT ATC ATA CCA		
*see*	F: TAC CAA TTA ACT TGT GGA TAG AC	170	[95]
	R: CTC TTT GCA CCT TAC CGC		
*seg*	F: CGT CTC CAC CTG TTG AAG G	327	[95]
	R: CCA AGT GAT TGT CTA TTG TCG		
*sei*	F: CAA CTC GAA TTT TCA ACA GGT AC	465	[95]
	R: CAG GCA GTC CAT CTC CTG		
*selj*	F: CAT CAG AAC TGT TGT TCC GCT AG	142	[95]
	R: CTG AAT TTT ACC ATC AAA GGT AC		

### 5.7. Statistical Analysis

The Pearson’s correlation (*r*) between the antimicrobial resistance phenotypes, resistance (*mecA* and *blaZ*), and virulence genes among the examined PVL-positive *S. aureus* was measured. For the resistance phenotype to each antimicrobial, the presence of a resistance and virulence gene received scores of 1, whereas susceptibility to antimicrobials and the absence of a resistance and virulence gene received scores of 0. The binary data (0/1) were then uploaded into R software (version 3.6.1; https://www.r-project.org, accessed on 17 September 2021). Using the package “corrplot”, the correlation at a significance of *p* < 0.05 was calculated using the “cor” and “cor.mtest” functions, and the plot was generated using the “corrplot” function. Based on the value of r, the degree of correlation was considered strong, moderate, and weak if the r value was >0.6, 0.4–0.6, and <0.4, respectively [98]. To visualize the overall distribution of the antimicrobial resistance phenotypes and virulence genes among the examined isolates, a heatmap with hierarchical clustering (Figure S2) based on the binary data (0/1) of antimicrobial resistance and virulence genes was created using the package “pheatmap” in R software (version 217 3.4.2) [99].

## Figures and Tables

**Table 1:** Frequency distribution of antimicrobial resistance and virulence genes among the examined PVL-positive *Staphylococcus aureus*.

Gene	Frequency Distribution		
	PVL-Positive MRSA (*n* = 37)	PVL-Positive MSSA (*n* = 76)	Total PVL-Positive *S. aureus* (*n* = 113)
*mecA*	37 (32.7%)	0	37 (32.7%)
*blaZ*	32 (28.3%)	64 (56.6%)	96 (84.9%)
*tst*	0	1 (0.9%)	1 (0.9%)
*hla*	18 (15.9%)	44 (38.9%)	62 (54.9%)
*sea*	20 (17.7%)	30 (26.6%)	50 (44.2%)
*seb*	2 (1.8%)	5 (4.4%)	7 (6.2%)
*sec*	0	1 (0.9%)	1 (0.9%)
*see*	0	0	0
*seg*	0	0	0
*sei*	0	0	0
*selj*	0	0	0
*sea/seb*	0	1 (0.9%)	1 (0.9%)

**Table 2:** Antimicrobial susceptibility of PVL-positive *Staphylococcus aureus* strains.

Antimicrobial Agent	Family	CPD	PVL-Positive MRSA (*n* = 37)		PVL-Positive MSSA (*n* = 76)		PVL-Positive *S. aureus* (*n* = 113)	
			Resistant No (%)	Sensitive No (%)	Resistant No (%)	Sensitive No (%)	Resistant No (%)	Sensitive No (%)
Penicillin (P)	β-lactam	10 μg	37 (32.7%)	0	65 (57.5%)	11 (9.7%)	102 (90.3%)	11 (9.7%)
Ampicillin(AMP)	β-lactam	10 μg	37 (32.7%)	0	43 (38.05%)	33 (29.2%)	80 (70.8%)	33 (29.2%)
Ciprofloxacin (CIP)	Fluoroquinolone	5 μg	24 (21.2%)	13 (11.5%)	23 (20.4%)	53 (46.9%)	47 (41.6%)	66 (58.4%)
Chloramphenicol (C)	Phenicols	30 μg	12 (10.6%)	25 (22.1%)	4 (3.5%)	72 (63.7%)	16 (14.0%)	97 (85.8%)
Clindamycin (DA)	Lincosamide	2 μg	20 (17.7%)	17 (15.04%)	22 (19.5%)	54 (47.8%)	42 (37.2%)	71 (62.8%)
Erythromycin (E)	Macrolide	15 μg	22 (19.5%)	15 (13.3%)	17 (15.04%)	59 (52.2%)	39 (34.5%)	74 (65.5%)
Gentamicin (CN)	Aminoglycoside	10 μg	11 (9.7%)	26 (23%)	12 (10.6%)	64 (56.6%)	23 (20.4%)	90 (79.6%)
Tetracycline (TE)	Tetracycline	30 μg	32 (28.3%)	5 (4.4%)	22 (19.5%)	54 (36.3%)	54 (47.79%)	59 (40.7%)
Trimethoprim- sulfamethoxazole (SXT)	Sulphonamide	25 μg	35 (31.0%)	2 (1.77%)	27 (23.9%)	49 (43.6%)	62 (54.9%)	51 (45.13%)

No: number.

**Table 3:** Antimicrobial resistance patterns and antibiotypes of PVL-positive MRSA strains.

Antibiotypes	Resistance Pattern	Isolate No (%) (*n* = 37)	MAR Index	MAR (*n* = 37)
I	P, AMP, C, and TE	1 (2.7%)	0.44	+
II	P, AMP, SXT, and TE	1 (2.7%)	0.44	+
III	P, AMP, CIP, and CN	1 (2.7%)	0.44	+
IV	P, AMP, C, SXT, and TE	4 (10.8%)	0.56	+
V	P, AMP, DA, E, and SXT	1 (2.7%)	0.56	+
VI	P, AMP, CIP, SXT, and TE	5 (13.5%)	0.56	+
VII	P, AMP, C, DA, SXT, and TE	1 (2.7%)	0.67	+
VIII	P, AMP, CIP, C, SXT, and TE	1 (2.7%)	0.67	+
IX	P, AMP, DA, E, SXT, and TE	2 (5.4%)	0.67	+
X	P, AMP, E, CN, SXT, and TE	1 (2.7%)	0.67	+
XI	P, AMP, CIP, DA, SXT, and TE	1 (2.7%)	0.67	+
XII	P, AMP, CIP, DA, E, SXT, and TE	7 (18.9%)	0.78	+
XIII	P, AMP, C, DA, E, SXT, and TE	1 (2.7%)	0.78	+
XIV	P, AMP, CIP, E, CN, SXT, and TE	1 (2.7%)	0.78	+
XV	P, AMP, CIP, DA, E, CN, and SXT	3 (8.1%)	0.78	+
XVI	P, AMP, DA, E, CN, SXT, and TE	1 (2.7%)	0.78	+
XVII	P, AMP, CIP, C, E, SXT, and TE	1 (2.7%)	0.89	+
XVIII	P, AMP, CIP, C, E, CN, SXT, and TE	1 (5.4%)	0.89	+
XIX	P, AMP, CIP, DA, E, CN, SXT, and TE	1 (2.7%)	0.89	+
XX	P, AMP, CIP, C, DA, E, CN, SXT, and TE	2 (5.4%)	1	+

**Table 4:** Antimicrobial resistance patterns and antibiotypes of PVL-positive MSSA strains.

Antibiotypes	Resistance Pattern	Isolate No (%) (*n* = 76)	MAR Index	MAR (*n* = 29)
I	0	5 (6.58%)	0.00	-
II	P	26 (34.2%)	0.11	-
III	CIP	1 (1.3%)	0.11	-
IV	AMP	3 (3.9%)	0.11	-
V	P and AMP	6 (7.9%)	0.22	-
VI	P and DA	1 (1.3%)	0.22	-
VII	AMP and DA	2 (2.6%)	0.22	-
VIII	P, AMP, and SXT	1 (1.3%)	0.33	-
IX	P, AMP, and CIP	2 (2.6%)	0.33	-
X	P, AMP, CIP, and DA	2 (2.6%)	0.44	+
XI	P, AMP, SXT, and TE	4 (5.2%)	0.44	+
XII	P, AMP, C, TE, and CIP	1 (1.3%)	0.56	+
XIII	P, AMP, CIP, CN, and SXT	1 (1.3%)	0.56	+
XIV	P, AMP, CIP, SXT, and TE	1 (1.3%)	0.56	+
XV	P, AMP, E, SXT, and TE	1 (1.3%)	0.56	+
XVI	P, AMP, CIP, C, and SXT	1 (1.3%)	0.56	+
XVII	P, AMP, CN, E, SXT, and TE	1 (1.3%)	0.67	+
XVIII	P, AMP, CIP, DA, E, and SXT	3 (3.9%)	0.67	+
XIX	P, AMP, CN, DA, SXT, and TE	2 (2.6%)	0.67	+
XX	P, AMP, CIP, DA, E, SXT, and TE	2 (2.6%)	0.78	+
XXI	P, AMP, DA, E, CN, SXT, and TE	1 (1.3%)	0.78	+
XXII	P, AMP, CIP, C, DA, E, SXT, and TE	2 (2.6%)	0.89	+
XXIII	P, AMP, CIP, DA, E, CN, SXT, and TE	7 (9.1%)	0.89	+

## Data Availability

The datasets generated during and/or analyzed during the current study are available from the corresponding author on reasonable request.

## Supplementary Materials

The following are available online at https://www.mdpi.com/article/10.3390/toxins14020097/s1, Figure S1: Association between resistance phenotypes, resistant genes, and virulence-associated genes in Panton-Valentine Leukocidin (PVL)-positive *Staphylococcus aureus* showing a significant correlation. The blue and orange colors of boxes indicate positive and negative correlation, respectively. The strength of the colors corresponds to the numerical value of the correlation coefficient (r); Figure S2: A heatmap showing the distribution of resistance phenotypes and genotypes, and virulence-associated genes in Panton-Valentine Leukocidin (PVL)-positive *Staphylococcus aureus*; Table S1: Virulence gene profiles and genotypic profiles of β-lactam resistance among Panton-Valentine Leukocidin (PVL)-positive *Staphylococcus aureus* strains.

## Abbreviations

PVL	Panton-Valentine leucocidin
MRSA	methicillin-resistant *S. aureus*
MSSA	methicillin-sensitive *S. aureus*
CLSI	Clinical and Laboratory Standards Institute
PCR	Polymerase chain reaction
TSB	Trypticase soy broth
MDR	Multidrug resistance
SEs	Staphylococcal enterotoxin genes
bp	Base pairs
TSST-1	Toxic shock syndrome toxin-1
VRSA	Vancomycin-resistant *S. aureus* strains
SFP	Staphylococcal food poisoning
CIP	Ciprofloxacin
E	Erythromycin
CN	Gentamicin
P	Penicillin
CL	Clindamycin
OX	Oxacillin
SAM	Ampicillin-sulbactam
C	Chloramphenicol
SXT	Trimethoprim-sulfamethoxazole
VA	Vancomycin Appendix A
