# Supplementary material for: Prevalence and Characterization of PVL-Positive Staphylococcus aureus Isolated from Raw Cow’s Milk
Source: Toxins (Basel). 2022 Jan 25;14(2):97. doi: 10.3390/toxins14020097 (PMC8876356; doi:10.3390/toxins14020097)
Supplement: Supplementary file 1 [file toxins-14-00097-s001.zip › toxins-1541937-supplementary.pdf]

toxins

MDPI

Supplementary Materials: Prevalence and Characterization of PVL-Positive *Staphylococcus aureus* Isolated from Raw Cow's Milk

Asmaa Sadat, Radwa Reda Shata, Alshimaa M. M. Farag Hazem Ramadan, Adel Alkhedaide, Mohamed Mohamed Soliman, Mohamed Elbadawy, Amira Abugomaa and Amal Awad

Table S1. Virulence gene profiles and genotypic profiles of β-lactam resistance among Panton-Valentine Leukocidin (PVL)-positive *Staphylococcus aureus* strains.

Genotype	Gene pattern	No. of isolates (%)
I	*pvl*	1 (0.88%)
II	*pvl, blaZ*	13 (11.5%)
III	*pvl, hla*	5 (4.4%)
IV	*pvl, mecA*	1 (0.88%)
V	*pvl, sea*	3 (2.65%)
VI	*pvl, blaZ, hla*	23 (20.35%)
VII	*pvl, blaZ, sea*	13 (11.5%)
VIII	*pvl, blaZ, seb*	2 (1.77 %)
IX	*pvl, hla, sea*	2 (1.77 %)
X	*pvl, mecA , hla*	3 (2.65%)
XI	*pvl, mecA, sea*	1 (0.88%)
XII	*pvl, mecA, blaZ*	4 (3.54%)
XIII	*pvl, mecA, blaZ, sea*	11 (9.7%)
XIV	*pvl, mecA, blaZ, hla*	6 (5.31%)
XV	*pvl, mecA, blaZ, seb*	1 (0.88%)
XVI	*pvl, mecA, blaZ, sec*	1 (0.88%)
XVII	*pvl, mecA, hla, sea*	1 (0.88%)
XVIII	*pvl, blaZ, hla, sea*	11 (9.7%)
XIX	*pvl, blaZ, hla, seb*	2 (1.77 %)
XX	*pvl, blaZ, hla, sea, seb*	1 (0.88%)
XXI	*pvl, mecA, blaZ, hla, sea*	6 (5.31%)
XXII	*pvl, mecA, blaZ, hla, seb*	1 (0.88%)
XXIII	*pvl, mecA, blaZ, hla, sea, tst*	1 (0.88%)

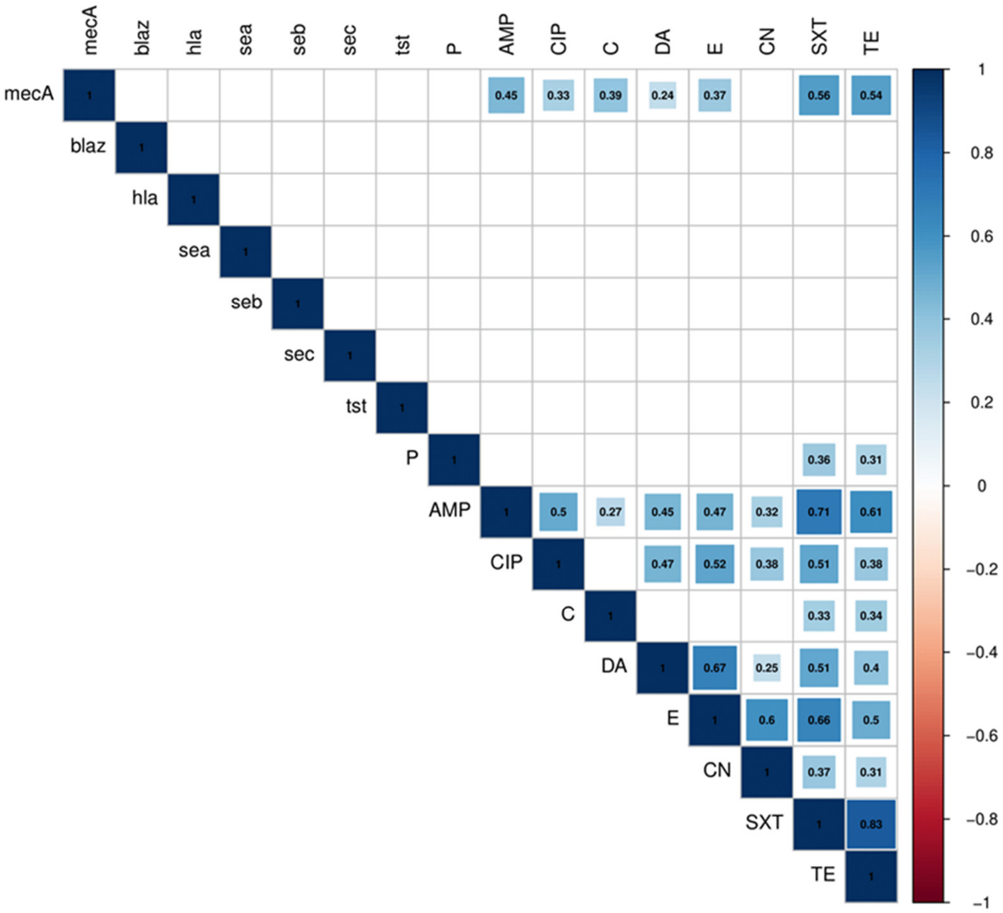

Figure S1. Association between resistance phenotypes, resistant genes, and virulence-associated genes in Panton-Valentine Leukocidin (PVL)-positive *Staphylococcus aureus* showing a significant correlation. The blue and orange colors of boxes indicate positive and negative correlation, respectively. The strength of the colors corresponds to the numerical value of the correlation coefficient (r). Hla: alpha-hemolysin encoding gene blaZ: penicillinase encoding gene mecA: penicillin-binding protein (PBP2-a) encoding gene and accounts for methicillin-resistance tst: toxic shock syndrome toxin TSST-1encoding gene. sea, seb, sec, see, Seg, sei, selj: Staphylococcus enterotoxins encoding genes nuc: *S. aureus*-specific thermonuclease encoding gene P: Penicillin AMP: Ampicillin CIP: Ciprofloxacin C: Chloramphenicol DA: Clindamycin E: Erythromycin CN: Gentamicin TE: Tetracycline SXT: Trimethoprim-sulfamethoxazole

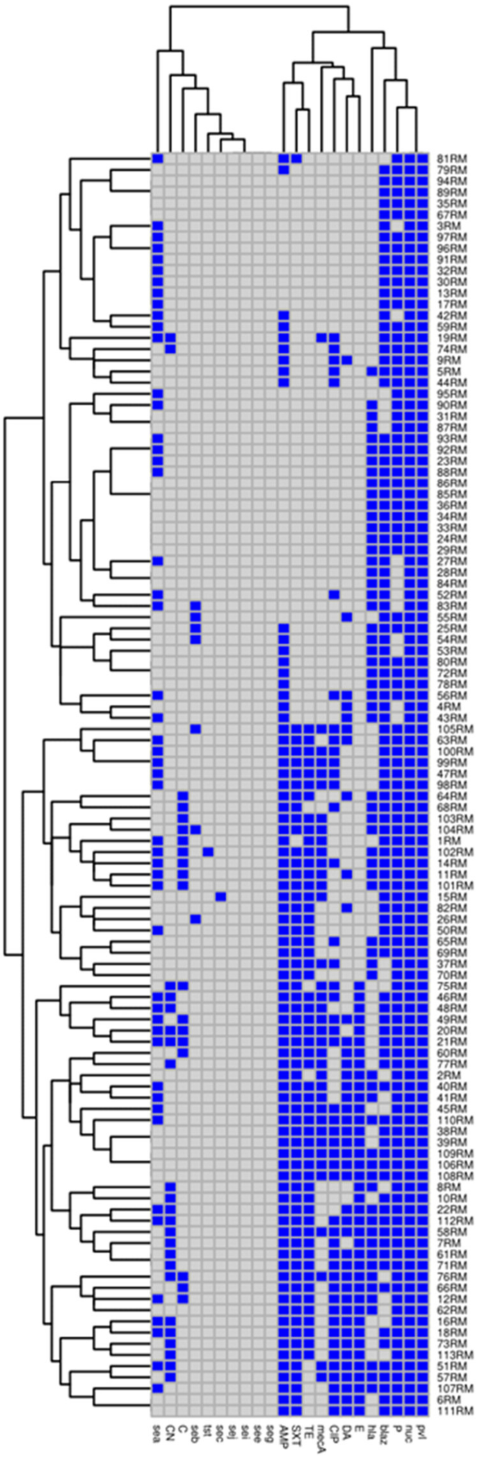

Figure S2. A heatmap showing the distribution of resistance phenotypes and genotypes, and virulence-associated genes in Panton-Valentine Leukocidin (PVL)-positive *Staphylococcus aureus*.
